# Misfit-induced changes of lattice parameters in two-phase systems: coherent/incoherent precipitates in a matrix

**DOI:** 10.1107/S1600576715022608

**Published:** 2016-02-01

**Authors:** Maryam Akhlaghi, Tobias Steiner, Sai Ramudu Meka, Eric Jan Mittemeijer

**Affiliations:** a Max Planck Institute for Intelligent Systems (formely MPI for Metals Reseach), Heisenbergstrasse 3, Stuttgart, 70569, Germany; b Robert Bosch GmbH Heat Treatment Processes and Heat Treatment Technology (CR/APM4), Postbox 300240, Stuttgart, 70442, Germany; c Institute for Materials Science, University of Stuttgart, Heisenbergstrasse 3, Stuttgart, 70569, Germany

**Keywords:** lattice-parameter change, precipitation-induced misfit, thermally induced misfit, elastic/plastic misfit accommodation, coherent/incoherent diffraction

## Abstract

Elastic accommodation of precipitation-induced or thermally induced misfit leads to lattice-parameter changes in crystalline multi-phase systems. Formulae for calculation of such misfit-induced lattice-parameter changes are presented for the aggregate (matrix + second-phase particles) and for the individual matrix and second phase, recognizing the occurrence of either coherent or incoherent diffraction by the matrix and second-phase particles. An overview and an (re)interpretation on the above basis is presented of published lattice-parameter data, obtained by X-ray diffraction analyses of aggregates of matrix plus second-phase particles. Examples for three types of systems consisting of a matrix with misfitting second-phase particles are dealt with, which differ in the origin of the misfit (precipitation or thermally induced) and in the type of diffraction (coherent or incoherent diffraction of matrix plus second-phase particles). The experimental data are shown to be in good to very good agreement with predictions according to the current treatment.

## Abstract 2

The article discusses misfit-induced lattice-parameter changes in two-phase systems as exposed by coherent diffraction of the assembly or incoherent diffraction by the matrix and the second-phase particles.

## 1. Introduction: micro- and macrostrains; coherent and incoherent diffraction   

The complex interplay of the chemical Gibbs energy change driving solid-state phase transformations and the deformation energy associated with the elastic/plastic accommodation of the misfit of parent and product phases hinders the prediction of the course and the kinetics of phase transformations (Starink *et al.*, 1993 ▸; Mittemeijer, 2010 ▸; Song *et al.*, 2014 ▸). Accordingly, unusual non-equilibrium phenomena can occur: occurrence of metastable phases, non-monotonic variation of transformation rate and unusual morphologies (Thompson *et al.*, 1994 ▸; Biglari *et al.*, 1995 ▸; Sennour & Esnouf, 2003 ▸; Liu *et al.*, 2003 ▸; Meka *et al.*, 2013 ▸; Loewy *et al.*, 2014 ▸; Villa *et al.*, 2014 ▸). Therefore, it is essential to acquire a fundamental understanding of the effects of developing misfit-strain energies on the thermodynamics and kinetics of phase transformations. Only by doing so can the resulting microstructure and properties of engineering components be optimized (Abbaschian *et al.*, 2010 ▸; Porter & Easterling, 1982 ▸; Guo & Sha, 2005 ▸). Thus the evolution of shape changes during precipitate growth in an elastically anisotropic, infinite matrix has been investigated for different two-phase systems [*e.g.* nickel-based alloys (Wang & Khachaturyan, 1995 ▸; Ratel *et al.*, 2006 ▸, 2010 ▸; Ardell, 2014 ▸) and piezoelectric piezomagnetic composites (Ni & Khachaturyan, 2007 ▸)].

The elastic strain field in the matrix surrounding very small (of the order of 1 nm) misfitting particles/point defects can be investigated by high-resolution transmission electron microscopy and by X-ray diffraction and diffuse scattering (static Debye–Waller factor; Krivoglaz, 1969 ▸). Larger sized misfitting particles can be associated with more extended misfit-strain fields, which evoke contrast in diffraction-contrast images recorded by transmission electron microscopy (Bor *et al.*, 2002 ▸) and broadening of diffraction lines [van Mourik *et al.*, 1988 ▸; van Berkum *et al.*, 1996 ▸; see in particular ch. 4 of Mittemeijer & Welzel (2012 ▸)].

The application of continuum elasticity theory to describe the elastic accommodation of the misfit of the (parent) matrix and the (product) second-phase particles (Eshelby, 1954 ▸, 1956 ▸; Khachaturyan, 1983 ▸) allows the calculation of microstrains, say the variance of the local strain 〈*e* ^2^〉 (van Mourik *et al.*, 1988 ▸; van Berkum *et al.*, 1996 ▸; Mura, 1982 ▸), and of the average (hydrostatic) macrostrain 〈*e*〉 (van Mourik *et al.*, 1983 ▸). The microstrain reveals itself through (extensive) diffraction-line broadening. The hydrostatic macroscopic strain causes a (distinct) change of the (average) lattice constant of a phase. Countless observations of diffraction-line broadening due to microstrain have been reported; in fact, analysis of diffraction-line broadening has become a standard method of microstructural characterization (Mittemeijer & Welzel, 2012 ▸). However, little experimental work has been devoted to the change of the (average) macroscopic lattice parameter by misfit strain (Mittemeijer *et al.*, 1981 ▸; Mittemeijer & van Gent, 1984 ▸; Zhou *et al.*, 1991 ▸; Müller *et al.*, 1997 ▸; Timmermans & Froyen, 1999 ▸; Chechenin *et al.*, 2002 ▸; Bruno *et al.*, 2003 ▸; Lu *et al.*, 2007 ▸). Against the above background, this manuscript presents a review of misfit-induced (average, macroscopic) lattice-parameter changes, as predicted and as observed. In this context in particular the distinction of cases of coherent and incoherent diffraction by second-phase/matrix assemblies is emphasized.

The coherent or incoherent nature of the diffraction by a second phase (such as precipitate particles) with respect to the diffraction by the matrix needs not coincide with the occurrence of a coherent or incoherent nature of the interfaces between the second-phase particles and the matrix. The coherency of diffraction depends not only on the specimen microstructure but also on the diffraction conditions, such as the length of the diffraction vector (van Berkum *et al.*, 1996 ▸; Rafaja *et al.*, 2004 ▸). Coherency/incoherency of diffraction depends on whether or not constructive interference of waves scattered by separate parts of the diffracting material (matrix and second phase) occurs. For a detailed discussion on the origin of coherent and incoherent diffraction of a second-phase particle and the matrix the reader is referred to van Berkum *et al.* (1996 ▸) [see also Mittemeijer (2010 ▸)]. The effect of lattice-parameter variation/the distribution of lattice spacings in a diffracting material on the occurrence of coherent or incoherent diffraction (and intermediates thereof) is considered most generally by Leineweber & Mittemeijer (2010 ▸).

Provided the matrix and second-phase particles diffract independently, *i.e.* incoherent diffraction of the matrix and of the second-phase particles occurs, the changes of the lattice parameters of the matrix and of the second-phase particles can be determined from measurements of the positions of the (separate) X-ray diffraction peaks of the matrix and of the second phase. If a coherent nature of the second-phase particles/matrix interfaces prevails, then, depending on the length of the diffraction vector and the extent of misfit-strain variation, coherent diffraction by second-phase particles and the matrix can occur.

## 2. Lattice-parameter changes due to elastically accommodated misfit   

The continuum theory for the fully elastic accommodation of the misfit of a point imperfection in a matrix was originally developed by Eshelby (1954 ▸, 1956 ▸). This theory is more likely to be applicable to the case of precipitation of misfitting entities of larger dimensions, such as second-phase particles (*i.e.* a block of atoms), which are more likely to exhibit elastic characteristics as for bulk materials. The formulae presented below describe the changes of the relative volume/lattice parameters of the matrix, of the misfitting particles and of the assembly, *i.e.* matrix plus misfitting particles [for more information see Akhlaghi *et al.* (2015 ▸)].

### 2.1. Change of the lattice parameter of the matrix   

For the case of an isotropic cubic finite matrix A with a (strain-free) lattice parameter 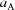, the change of lattice parameter, 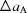, of the finite matrix due to the introduction of misfitting isotropic inclusions B can be written as (Christian, 2002 ▸; Mittemeijer *et al.*, 1981 ▸)
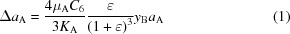
with
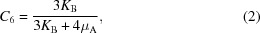
where 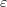 represents the linear misfit parameter (see below), 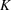 and 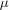 represent the bulk modulus and the shear modulus, respectively, and 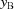 is the volume fraction of inclusions. It is essential to recognize that the matrix is of finite dimensions (Akhlaghi *et al.*, 2015 ▸).

### 2.2. Change of the lattice parameter of the misfitting phase   

For the case of isotropic second-phase particles of cubic crystal structure with a (strain-free) lattice parameter 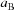, the change of the lattice parameter, 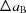, of the misfitting second phase in a finite matrix is (van Mourik *et al.*, 1985 ▸)
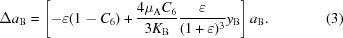



### 2.3. Change of the lattice parameter of the assembly (matrix plus misfitting phase)   

For a finite aggregate of cubic crystal structure and a (strain-free) lattice parameter of the matrix, *a*, the resulting change of the lattice parameter, 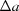, of the aggregate for a volume fraction 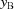 of inclusions B is given by (Christian, 2002 ▸)
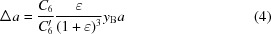
with
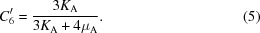



### 2.4. The misfit parameter   

The linear misfit parameter appearing in the above equations can be given an explicit analytical expression in the following cases:

(i) Thermal misfit. This misfit originates from a change in the temperature of the two- (or multi-)phase composite (matrix + second-phase particles) and is due to the difference in linear thermal expansion coefficients of the matrix (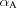) and the second-phase precipitate (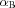). This type of misfit can be described by the linear thermal misfit parameter, 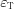:
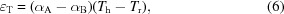
where 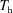 and 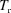 stand for the elevated heat-treatment temperature and the considered low (*e.g.* room) temperature, where measurements are made, respectively.

(ii) Precipitation-induced misfit. This misfit originates from the different specific volumes of (solute and solvent) atoms in the second phase (precipitate) and the (solute and solvent) atoms in the matrix. This type of misfit can be described by the linear volume misfit parameter, 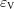:
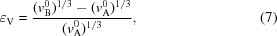
where 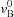 and 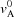 are the volume of the strain-free precipitate particle and the volume occupied by the atoms of the precipitate particle as previously incorporated in the matrix, respectively.

## 3. Observations and discussion   

At elevated (aging/annealing) temperature (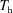), development of a misfitting second phase can occur from a supersaturated matrix. The volume misfit (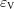) of the two phases can initially be accommodated elastically under preservation of a coherent interface. Upon prolonged annealing, leading to an increasing size of the second-phase particles, this misfit can no longer be accommodated fully elastically, and the interface can become semi or fully incoherent (Porter & Easterling, 1982 ▸; Quek *et al.*, 2011 ▸; Meka *et al.*, 2013 ▸; Geslin *et al.*, 2014 ▸).

Upon cooling from elevated temperature (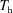) to room temperature (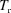), irrespective of whether the precipitation-induced misfit at elevated temperature is accommodated elastically or plastically, thermal misfit arises which has to be accommodated either elastically (Mittemeijer *et al.*, 1981 ▸) or plastically (van Berkum *et al.*, 1991 ▸). In many cases, upon fast cooling of the specimens, the thermal misfit accommodation occurs mainly elastically (even if an incoherent matrix/second-phase particle occurs), and it can remain in that state for a long time owing to very slow rates of plastic relaxation processes at low (room) temperature.

As long as the (transformation or thermal) misfit is accommodated elastically, its effect on the expansion/contraction of the system consisting of the matrix and misfitting second-phase particles can be quantified by considering the model description presented in §[Sec sec2]2. Thus, in the following, three types of system consisting of a matrix with misfitting second-phase particles are dealt with (see Table 1 ▸), which differ in the origin of misfit (transformation induced or thermally induced) and in the type of diffraction (coherent or incoherent diffraction of matrix plus second-phase particles). For the different systems described in the following, the values of the constants and misfit parameters used for the calculation of lattice-parameter changes of the matrix [equation (1)], the precipitate [equation (3)] and the assembly [equation (4)] are presented in Tables 2 ▸ and 3 ▸, respectively.

### 3.1. Coherent diffraction by the assembly matrix plus second-phase particles: transformation misfit   

#### 3.1.1. *Misfitting coherent alloying element nitrides in ferrite*   

Upon nitriding of ferritic Fe–Me (Me = Cr, V, Ti) alloys, tiny nitride precipitates (MeN) develop (Schacherl *et al.*, 2002 ▸; Miyamoto *et al.*, 2006 ▸; Vives Díaz *et al.*, 2008 ▸; Jack, 1976 ▸; Rickerby *et al.*, 1986 ▸). At least initially these nitrides possess coherent interfaces with the matrix. Matrix/nitride-particle misfit in this system, as considered at room temperature, originates from (i) the specific volume misfit induced by nitride precipitation from a supersaturated matrix as a consequence of elastic accommodation of the misfit while the precipitate/matrix interface remains coherent and (ii) the thermal misfit induced by cooling after nitriding. The specific volume misfit between the MeN precipitates and the ferrite matrix can be calculated from equation (7), where 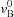 and 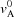 can now be taken as the molar volumes of the MeN precipitates and the matrix, respectively. After complete precipitation, the molar volume of the MeN precipitates can be calculated from the volume of the precipitate unit cell divided by the number of metal atoms occupying one unit cell of the precipitate, and the molar volume of the matrix (in this case, containing only Fe atoms) can be calculated from the volume of the matrix unit cell divided by the number of iron atoms occupying one unit cell of the matrix. In this case the thermal misfit (corresponding to the difference of the thermal expansion coefficients of the Fe matrix and the developed nitrides) is negligible as compared to the precipitation-induced misfit (*i.e.* 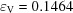 and 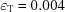 for cooling from 673 K to room temperature for nitrided Fe–Cr alloy and 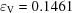 and 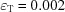 for cooling from 673 to 298 K for nitrided Fe–V alloys; Basinski *et al.*, 1955 ▸; Samsonov, 1964 ▸).

**Figure 1:**
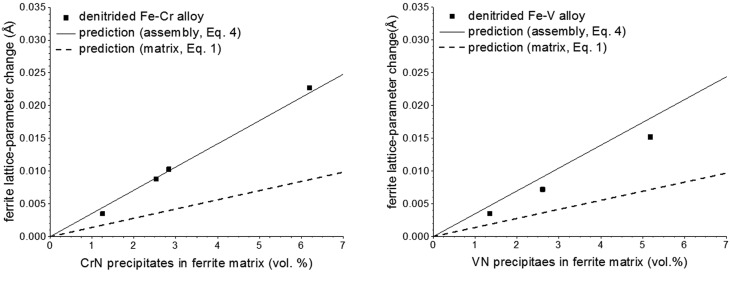
Experimental ‘ferrite’ lattice-parameter change [dots; data from Akhlaghi *et al.* (2015 ▸)] as a function of vol.% of (*a*) CrN precipitates and (*b*) VN precipitates and the predictions on the basis of equation (4) (solid line) and equation (1) (dashed line). The error in the experimental lattice-parameter values obtained after fitting the measured diffractograms is ±0.0003 Å.

This system is an example of coherent diffraction by the entity matrix plus precipitates in the presence of coherent interfaces between the Fe matrix [of body-centred cubic (b.c.c.) crystal structure] and the MeN precipitates (of NaCl-type crystal structure) . Hence, the overall expansion of the assembly (composed of MeN nitride precipitates and ferrite matrix), expressed in terms of the change in the lattice parameter deduced from the position of the ‘ferritic’ peak maxima, can be calculated using equation (4) as a function of the volume fraction of alloying element nitrides. Experimental values for such changes of lattice parameter upon nitriding of Fe–Cr and Fe–V alloys were obtained by employing X-ray diffraction on homogenously nitrided (thus macroscopically strain-free) Fe–Me specimens (Akhlaghi *et al.*, 2015 ▸).^1^ The volume fraction of (CrN and VN) nitride precipitates was determined (by weight-change measurements) from the N content of denitrided thin foils [*i.e.* after removing from the specimen, by denitriding, the dissolved (excess) N and the excess N adsorbed at the nitride-platelet faces during nitriding; Somers *et al.*, 1989 ▸, Podgurski & Davis, 1981 ▸]. The volume fraction of precipitates was varied by varying the nitriding time (for times larger than the minimal time to achieve a homogenously nitrided specimen) and/or varying the amount of Me (Cr, V) in the alloy (realizing full precipitation throughout the specimen). The results are shown in Fig. 1 ▸ (the single dots). The predictions on the basis of equation (4) (solid lines in Fig. 1 ▸) are in good agreement with the experimental data. Evidently, in this case equation (1) (implying incoherent diffraction of matrix and precipitates; dashed lines in Fig. 1 ▸) does not at all correctly predict the ferritic (X-ray) lattice-parameter changes observed for the nitrided Fe–Cr and Fe–V alloys.

#### 3.1.2. *Misfitting coherent cobalt precipitates in copper*   

A thin film of a metastable Cu–Co solid solution can be prepared by co-deposition of Cu and Co by magnetron sputtering up to a maximum of 12 at.% Co. For Co contents larger than 12 at.%, clusters/tiny precipitates of Co occur in the alloy (Berkowitz *et al.*, 1992 ▸; Michaelsen, 1995 ▸).

The X-ray diffraction patterns recorded from such Cu–Co alloy films of different Co concentrations show the occurrence of a single face-centred cubic (f.c.c.) Cu(Co) phase up to 65 at.% Co. Only beyond this Co concentration can additional hexagonal close-packed (Co) reflections be observed [see Fig. 5 of Michaelsen (1995 ▸)]. Evidently, the Co inclusion/particles present in the Cu–Co alloy films with more than 12 at.% Co and up to 65 at.% Co diffract coherently with the matrix.

**Figure 2:**
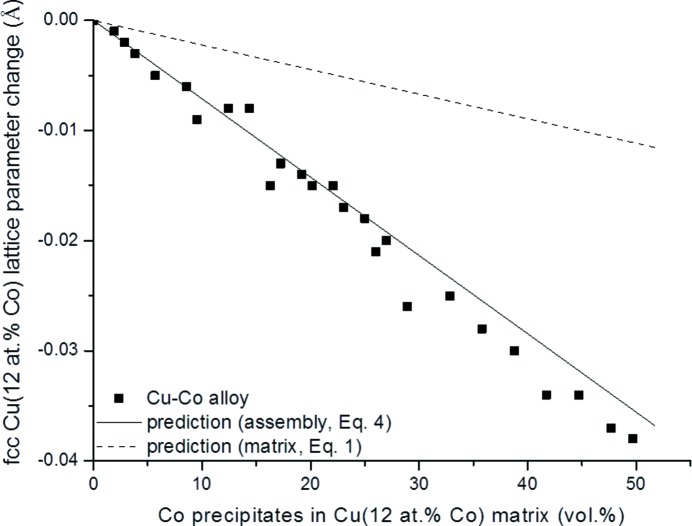
Experimental Cu–12 at.% Co lattice-parameter change [dots; data from Michaelsen (1995 ▸)] as a function of vol.% of Co precipitates in the Cu–12 at.% Co matrix and the predictions on the basis of equation (4) (solid line) and equation (1) (dashed line). For the application of equation (4), it is assumed that Co in the Cu matrix has precipitated as f.c.c. Co particles.

The experimentally obtained lattice parameters of the f.c.c. Cu(Co) films as a function of Co concentration are shown as single dots in Fig. 2 ▸ for Co concentrations between 12 and 65 at.%. The predictions of the lattice-parameter values of the aggregate Cu matrix (Cu 12 at.% Co solid solution) and second-phase Co particles as a function of the volume percentage of Co precipitates in the Cu–12 at.% Co matrix according to equation (4) (coherent diffraction of matrix and inclusions) and equation (1) (incoherent diffraction of matrix and inclusions) are also shown in Fig. 2 ▸. Evidently, the experimentally determined lattice-parameter values and the predictions on the basis of equation (4) (solid black line in Fig. 2 ▸) are in very good agreement, implying occurrence of coherent diffraction of matrix and precipitates. In this case predictions according to equation (1) (dashed line in Fig. 2 ▸) are not at all in agreement with the experimental data. The prediction of the lattice parameter of f.c.c. Cu(Co) can also be made on the basis of Vegard’s law [linear interpolation between Cu and f.c.c. Co lattice parameters as shown by Michaelsen (1995 ▸)].

### 3.2. Incoherent diffraction of the matrix and second-phase particles: transformation misfit   

#### 3.2.1. *Misfitting incoherent nitrides in ferrite*   

Upon nitriding of pure α-Fe (ferrite) in an atmosphere of a certain nitriding potential, N dissolves in the ferrite matrix. The formation of this solid solution leads to an increase of the lattice parameter of the ferrite matrix. By quenching after nitriding the solid solution can be retained. The system is then metastable. During aging of the α-Fe matrix supersaturated with dissolved N at room temperature, formation of α′′-Fe_16_N_2_ precipitates occurs in association with depletion of N from the ferrite matrix. Experimentally, a decrease of the ferrite lattice parameter is observed at room temperature during development of the α′′ precipitates (Mittemeijer *et al.*, 1980 ▸; Mittemeijer, 1981 ▸; Ferguson & Jack, 1983 ▸; Mittemeijer & van Gent, 1984 ▸). Upon prolonged aging, after complete precipitation of α′′, a constant lattice-parameter value is established that is larger than the one expected for pure α-Fe (the equilibrium solubility of N in α-Fe at room temperature is practically nil) [see Fig. 4 of Mittemeijer (1981 ▸), Fig. 1 of Mittemeijer *et al.* (1980 ▸) and Fig. 7 of Ferguson & Jack (1983 ▸)].

**Figure 3:**
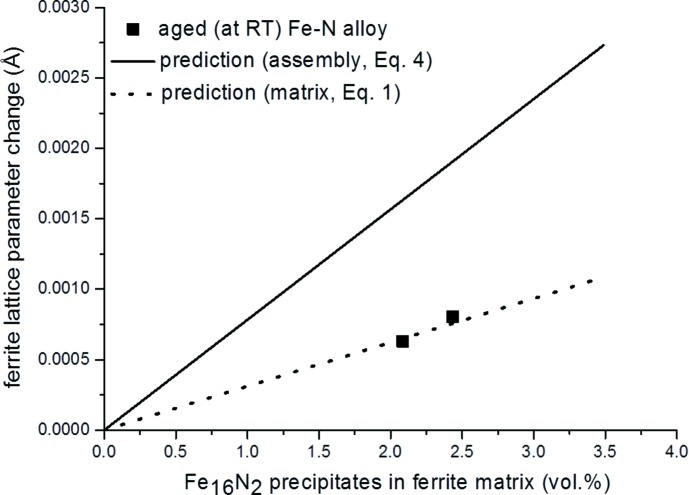
Experimental Fe-matrix lattice-parameter change [dots; data from Mittemeijer & van Gent (1984 ▸) and Ferguson & Jack (1983 ▸)] as a function of vol.% of α′′ precipitates and the predictions on the basis of equation (4) (solid line) and equation (1) (dotted line).

The values of the constant lattice parameter of the ferrite matrix at this stage of prolonged aging at room temperature, *i.e.* after all N has precipitated, as recorded from specimens containing different amounts of nitrogen, are shown as a function of the volume fraction of developed α′′ precipitates in Fig. 3 ▸. Predictions for the change of the ferritic lattice parameter are also shown in Fig. 3 ▸: (i) for the case of incoherent diffraction of matrix and precipitates [equation (1); dotted line in Fig. 3 ▸] and (ii) for the case of coherent diffraction of the assembly matrix plus precipitates [equation (4); full line in Fig. 3 ▸]. The experimental results very well agree with the predictions according to equation (1): evidently, in this case of prolonged ageing, the α-Fe matrix and the α′′-nitride precipitates diffract incoherently. The separate α′′ reflections are very weak,^2^ because of the small amounts of N (and thus α′′) in the specimens, and cannot be observed by conventional laboratory X-ray diffraction [as is the case here; synchrotron radiation would be required as shown by van Genderen *et al.* (1993 ▸)]. 

**Figure 4:**
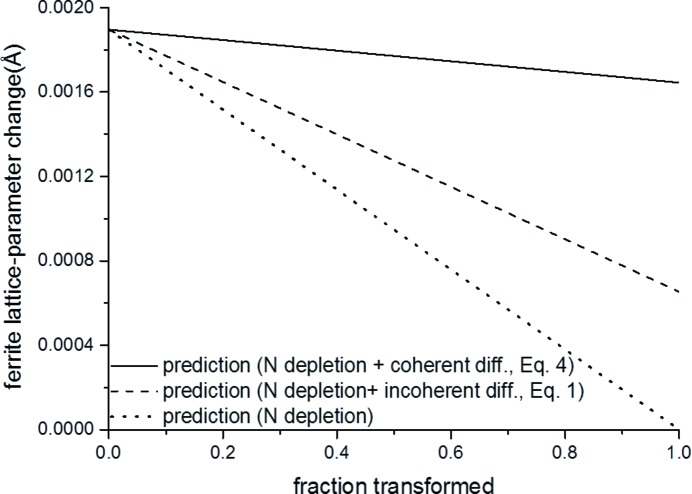
Lattice-parameter change of the nitrided ferrite matrix upon precipitation of α′′ as a function of the fraction of N precipitated for a specimen of ferrite containing 0.24 at.% N. Calculations on the basis of equation (4) (solid line) and equation (1) (dashed line), considering the effect of nitrogen depletion of the ferrite matrix. The change of the ferrite lattice parameter due to only N depletion is also shown [dotted line; calculated using equation (1) of Mittemeijer *et al.* (1980 ▸)].

As mentioned above, before complete precipitation of α′′, *i.e.* in the intermediate stages of aging, the lattice parameter of ferrite decreases continuously as the ferrite-lattice contraction by the depletion of N, dissolved on the interstitial (octahedral) sites of the ferrite matrix, is larger than the ferrite-lattice expansion due to the formation of misfitting α′′ precipitates diffracting either coherently or incoherently with the matrix. Thus, during precipitation of α′′, a (possibly occurring) change of the diffraction conditions from coherent to incoherent will only lead to a change in magnitude of the resulting lattice-parameter decrease. Corresponding calculations for the change of the ferritic lattice parameter as a function of the fraction transformed (*i.e.* the precipitated fraction of N), for a fixed N content of the specimen, are shown in Fig. 4 ▸: (i) for the case considering both the effect of the depletion of dissolved N from the solid solution [equation (1) of Mittemeijer *et al.* (1980 ▸)] and incoherent diffraction of matrix and precipitate [equation (1); dashed line in Fig. 4 ▸], (ii) for the case considering both the effect of the depletion of dissolved N from the solid solution [equation (1) of Mittemeijer *et al.* (1980 ▸)] and coherent diffraction of assembly matrix plus precipitates [equation (4); full line in Fig. 4 ▸], and (iii) for the case considering only the effect of the depletion of dissolved N from the solid solution [equation (1) of Mittemeijer *et al.* (1980 ▸); dotted line in Fig. 4 ▸]. Evidently, ignorance of the effect of misfit strain on lattice-parameter changes results in a strongly erroneous (too small) precipitated fraction of N as derived from the decrease of lattice parameter.

### 3.3. Incoherent diffraction of the matrix and second-phase particles: thermal misfit   

#### 3.3.1. *Misfitting incoherent silicon in aluminium*   

During high-temperature annealing/aging of an Al matrix supersaturated with dissolved Si, precipitation of incoherent Si occurs. Initially, lattice expansion of the Al matrix happens owing to partially elastic accommodation of the precipitate/matrix misfit [*cf*. equation (1)] and depletion of Si from the solid solution [see Fig. 9 of van Mourik *et al.* (1985 ▸)]. This same process also results in lattice contraction for the Si precipitates [*cf*. equation (3); see Fig. 7 of van Mourik *et al.* (1985 ▸)]. Upon prolonged aging at high temperature, this precipitation-induced misfit relaxes fully, *i.e.* is accommodated entirely plastically. Then a case of incoherent diffraction of matrix and precipitates in the presence of incoherent interfaces between the Al matrix (with f.c.c. crystal structure) and the Si precipitates (with diamond cubic crystal structure) is established.

**Figure 5:**
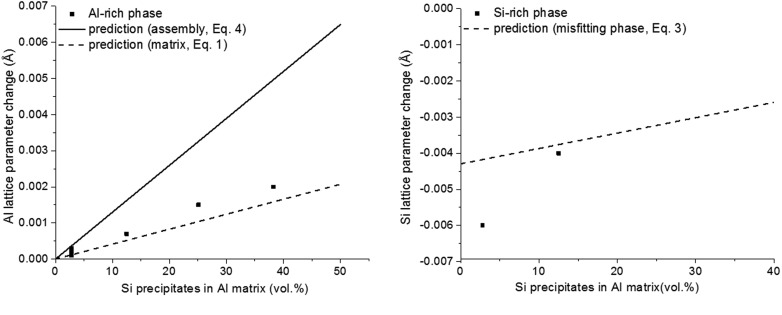
(*a*) Experimental Al-matrix lattice-parameter change [dots; data from Table 1 ▸; experiments were performed between 425 and 448 K by Mittemeijer *et al.* (1981 ▸)] as a function of vol.% of Si precipitates and the predictions on the basis of equation (4) (solid line) and equation (1) (dashed line). (*b*) Experimental Si-precipitate lattice-parameter change [dots; data from Table 1 ▸; experiments were performed at 425 K by van Mourik *et al.* (1985 ▸)] as a function of vol.% of Si precipitates and the prediction on the basis of equation (3) (dashed line). The experimental lattice-parameter values were determined with a precision of 1–2 parts in 40 000.

Now, upon rapid cooling of such a relaxed two-phase (pure Al matrix + incoherent Si particles) specimen to room temperature, thermal misfit develops, owing to the difference of the thermal expansion coefficients of the Al matrix and Si precipitates; this misfit (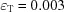 for cooling from 425 K to room temperature) is accommodated elastically. The changes of the lattice parameters of the matrix and the precipitates were measured as a function of the volume fraction of developed precipitates (Si), utilizing the separate diffraction peaks from the matrix and precipitates as recorded from fast-cooled (to suppress any relaxation of the thermal misfit during cooling) two-phase specimens of different Si contents. The thus experimentally obtained lattice-parameter change for the Al matrix is shown as a function of the volume fraction of Si precipitates in Fig. 5 ▸(*a*) (dots). The prediction (now) on the basis of equation (1) [dashed line in Fig. 5 ▸(*a*)] is in good agreement with the experimental data. Evidently, in this case equation (4) [implying coherent diffraction of matrix and precipitate; solid line in Fig. 5 ▸(*a*)] does not at all correctly predict the lattice-parameter change of the aluminium matrix. An only reasonable agreement occurs for the theoretical [equation (3)] and experimental values for the lattice-parameter change of the silicon precipitates (Fig. 5 ▸ *b*); significant experimental errors are inherent in the determination of the minority-phase lattice parameter of the Si precipitates, in particular for the alloy of the lowest Si content of 2.8 vol.% (*cf*. Fig. 5 ▸ *b*).

#### 3.3.2. *Misfitting incoherent alloying element nitrides in ferrite*   

As described in §3.1, upon nitriding Fe–Cr alloys, misfitting CrN particles develop which initially have a coherent interface with the matrix, leading to an overall lattice expansion as observed from the change of the ‘ferrite’ lattice parameter (*cf*. Fig. 1 ▸). Upon aging (at the nitriding temperature), coarsening of the precipitates occurs [see Fig. 5 of Steiner *et al.* (2015 ▸)] in association with the development of incoherent matrix/precipitate interfaces: plastic accommodation of the precipitation-induced misfit. Consequently, the system in such conditions exhibits incoherent diffraction of the matrix and precipitates [*i.e.* the matrix and coarsened precipitates now diffract separately: indeed both matrix and precipitate reflections are detected; see Fig. 11 of Steiner *et al.* (2015 ▸)].

Upon cooling of this relaxed two-phase composite (Fe matrix + incoherent CrN nitrides) to room temperature, thermal misfit develops, owing to the difference of the thermal expansion coefficients of the Fe matrix and CrN nitrides; this misfit (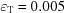 for cooling from 773 K to room temperature) gets accommodated elastically. The change of the lattice parameter of the ferrite matrix by elastic accommodation of this thermal misfit has been measured by X-ray diffraction and has been predicted on the basis of equation (1). A very good agreement occurs (Table 4 ▸). The change of the lattice parameter of the precipitates has also been measured by X-ray diffraction and has been predicted on the basis of equation (3). As predicted, the experimental change of the lattice parameter of the precipitates has a sign opposite to that of the matrix (*cf*. Table 4 ▸). The quantitative agreement here is less good, which can be ascribed to uncertainty concerning the strain-free lattice parameter of CrN (ICDD, 2002 ▸) and limited accuracy in the experimental determination of the lattice parameter of the minority phase.

## 4. Conclusions   

Formulae have been presented for the calculation of lattice-parameter changes induced in misfitting matrix/second-phase-particle systems.

Diffraction analysis of such lattice-parameter changes requires distinction of (i) coherent diffraction by the matrix/second-phase-particle aggregate and (ii) incoherent diffraction of the matrix and second-phase particles.

A number of examples presented for cases of precipitation-induced specific volume misfit and cooling-induced thermal misfit show good to very good agreement of theoretical predictions and experimental observations.

The discussed misfit-induced lattice-parameter/volume changes are often ignored in the diffraction (and also in the dilatometric) analysis of phase-transformation kinetics. The present treatment provides a direct route for correction of these effects. 

## Figures and Tables

**Table 1:** Types of system consisting of a matrix with misfitting second-phase particles, categorized on the basis of the type of elastically accommodated misfit (column 2) and of the type of precipitate/matrix diffraction (column 3; *cf*. §1) Equations to be used for the calculation of lattice-parameter changes of the assembly [equation (4)], the matrix [equation (1)] and the second-phase particle [equation (3)] (column 4) and system examples (column 5) are also shown.

Type of system	Type of elastically accommodated misfit	Type of precipitate/matrix diffraction	Lattice-parameterchange	Case studies
1	Transformation misfit	Coherent	Equation (4)	Nitrided Fe–Me alloys (Akhlaghi *et al.*, 2015 ▸), Co clusters in decomposed Cu–Co alloy (Michaelsen, 1995 ▸)
2	Transformation misfit	Incoherent	Equations (1) and (3)	Aged (at RT) Fe–N alloy (Mittemeijer & van Gent, 1984 ▸; Mittemeijer, 1981 ▸; Ferguson & Jack, 1983 ▸)
3	Thermal misfit	Incoherent	Equations (1) and (3)	Aged Al–Si alloy (Mittemeijer *et al.*, 1981 ▸; van Mourik *et al.*, 1985 ▸, 1988 ▸, 1983 ▸), nitrided and aged Fe–Cr alloys (Steiner *et al.*, 2015 ▸)

**Table 2:** Values of the physical constants used in the model calculations of lattice-parameter changes

	Lattice constants (Å)	*K* (GPa)	μ (GPa)	α × 10 ^−6^ (K^−1^)
Fe	2.8664 (b.c.c.) (ICDD, 2002 ▸)	166 (Wawra, 1978 ▸)	82 (Wawra, 1978 ▸)	12.1 (Smithells, 1976 ▸)
CrN	4.1400 (NaCl) (ICDD, 2002 ▸)	360 (Grossman *et al.*, 1999 ▸)	–	2.3 (Samsonov, 1964 ▸)
VN	4.1392 (NaCl) (ICDD, 2002 ▸)	300 (Dzivenko *et al.*, 2010 ▸)	–	8.1 (Samsonov, 1964 ▸)
Cu	3.6150 (f.c.c.) (ICDD, 2002 ▸)	140 (Wang *et al.*, 2009 ▸)	48 (Smithells, 1976 ▸)	17 (Smithells, 1976 ▸)
Co	3.5442 (f.c.c.) (Taylor & Floyd, 1950 ▸)	140† (Wang *et al.*, 2009 ▸)	–	–
Fe_16_N_2_	5.7200, 6.2900 (b.c.t.‡) (ICDD, 2002 ▸)	166† (Wawra, 1978 ▸)	–	–
Al	4.0494 (f.c.c.) (ICDD, 2002 ▸)	75 (Smithells, 1976 ▸)	26 (Smithells, 1976 ▸)	23.5 (Smithells, 1976 ▸)
Si	5.4309 (diamond) (ICDD, 2002 ▸)	98 (Madelung *et al.*, 2001 ▸)	–	3 (Roberts, 1981 ▸)

† The bulk modulus of f.c.c. cobalt precipitates is assumed to be the same as that of the Cu matrix and the bulk modulus of the Fe_16_N_2_ precipitates is considered to be the same as that of the Fe matrix.

‡ b.c.t. – body-centred tetragonal.

**Table 3:** Values of the misfit parameters used in the model calculations of lattice-parameter changes Values of thermal misfits are italic; the other values refer to precipitation-induced misfit.

	Matrix		
Inclusion	Fe	Cu	Al
CrN	0.1464	–	–
	*0.004 (Δ*T* = 648 K)*		
	*0.005 (Δ*T* = 748 K)*		
VN	0.1461	–	–
	*0.002 (Δ*T* = 648 K)*		
Co	–	−0.0173	–
Fe_16_N_2_	0.0299	–	–
Si	–	–	*0.003 (Δ*T* = 400 K)*

**Table 4:** Change of the Fe-matrix lattice parameter and that of the CrN precipitates for a homogenously nitrided (till saturation) Fe–4.5 at.% Cr specimen as measured after aging at 773 K for 78 h, and as predicted on the basis of equations (1) and (3), respectively

Phase	Δ*a* prediction (Å)	Δ*a* experiment (Å)
α-Fe	+0.0005 [equation (1)]	+0.0006 (3) (Steiner *et al.*, 2015 ▸)
CrN	−0.0045 [equation (3)]	−0.0020 (3) (Steiner *et al.*, 2015 ▸)

## Footnotes

1 No separate nitride reflections could be discerned; the strongly broadened ferritic peaks represent coherent diffraction by the distorted (by elastic misfit-strain accommodation) ferrite matrix and the nitride precipitates [for details see Akhlaghi *et al.* (2015 ▸)].

2 The α′′ crystal structure can be conceived as a superstructure developing in nitrogen ferrite by ordering of N on the sublattice of octahedral interstices. The intensity of the α′′-structure-specific superstructure reflection is governed by the weak scattering power of the N atoms.
